# Biomedical Applications of Carbon Nanomaterials: Fullerenes, Quantum Dots, Nanotubes, Nanofibers, and Graphene

**DOI:** 10.3390/ma14205978

**Published:** 2021-10-11

**Authors:** Manish Gaur, Charu Misra, Awadh Bihari Yadav, Shiv Swaroop, Fionn Ó. Maolmhuaidh, Mikhael Bechelany, Ahmed Barhoum

**Affiliations:** 1 Centre of Biotechnology, University of Allahabad, Prayagraj 211002, India; manishgaur@allduniv.ac.in (M.G.); charu.pharmacy0801@gmail.com (C.M.); 2 Department of Biochemistry, Central University of Rajasthan, Ajmer 305817, India; shivswaroop@curaj.ac.in; 3 National Centre for Sensor Research, School of Chemistry, Dublin City University, D09 V209 Dublin, Ireland; fionn.omaolmhuaidh2@mail.dcu.ie; 4 Institut Européen des Membranes (IEM), UMR 5635, University Montpellier, ENSCM, CNRS, Place Eugène Bataillon, 34095 Montpellier, France; 5 Nano Struc Research Group, Chemistry Department, Faculty of Science, Helwan University, Cairo 11795, Egypt; 6 School of Chemical Sciences, Fraunhofer Project Centre, Dublin City University, D09 V209 Dublin, Ireland

**Keywords:** drug delivery, biomedical scaffold, tissue engineering, wound healing, biosensing, bioimaging, vaccination, photodynamic therapy, bioavailability, cytotoxicity

## Abstract

Carbon nanomaterials (CNMs) have received tremendous interest in the area of nanotechnology due to their unique properties and flexible dimensional structure. CNMs have excellent electrical, thermal, and optical properties that make them promising materials for drug delivery, bioimaging, biosensing, and tissue engineering applications. Currently, there are many types of CNMs, such as quantum dots, nanotubes, nanosheets, and nanoribbons; and there are many others in development that promise exciting applications in the future. The surface functionalization of CNMs modifies their chemical and physical properties, which enhances their drug loading/release capacity, their ability to target drug delivery to specific sites, and their dispersibility and suitability in biological systems. Thus, CNMs have been effectively used in different biomedical systems. This review explores the unique physical, chemical, and biological properties that allow CNMs to improve on the state of the art materials currently used in different biomedical applications. The discussion also embraces the emerging biomedical applications of CNMs, including targeted drug delivery, medical implants, tissue engineering, wound healing, biosensing, bioimaging, vaccination, and photodynamic therapy.

## 1. Introduction

CNMs are an emerging field of nanomaterials that offer a propitious approach in drug delivery, tissue regeneration, medical implants, and other applications [1]. CNMs are defined as materials with sizes ranging from 1–100 nm. As a whole, nanotechnology has become a promising field, which has revolutionized the cure and diagnosis of diseases; this is due to the development of CNM-based materials with potential applications for disease cure and diagnosis [2]. CNMs possess unique properties that can be tuned through production methods to enhance characteristics such as optical activity, multifunctional surface morphology, surface area, drug loading efficacy, biocompatibility, and immunogenicity. This high degree of control over several key characteristics of the material offers advantages over metal-based biomaterials, such as titanium. A higher degree of freedom when engineering the material allows for more sophisticated applications, such as highly controlled drug release and drug delivery [3]. There are several types of CNMs: fullerene (or Buckyball), nano-diamond, amorphous nanocarbon, graphene nanosheets, graphene oxide (GO), single-walled carbon nanotube (SWCNTs), multi-walled carbon nanotube (MWCNTs), graphene quantum dots (GQDs), and carbon foam [4]. CNM families feature unique characteristics that can be applied to diverse biological applications [5]. They also offer potential anti-cancer and anti-inflammatory properties [6].

Recent studies have indicated that there are various reasons and factors responsible for the toxicity of CNMs. The size and shape, as well as the aspect ratio, of CNMs all affect their toxicity upon cellular uptake, interfering with cellular processes both in the cytoplasm and in the nucleus. Reports also suggest [7] that CNMs are subject to adulteration or contamination by certain substances, such as metal ions, during synthesis; these substances also contribute to the overall cytotoxicity of the final product materials. CNMs can damage lipids and DNA when they are taken up by cancer cells as they stimulate reactive oxygen species (ROS) and cause cell death [8]. Similarly, increasing ROS levels by graphene materials affects the metabolic activity of the macrophages and damages the mitochondrial membrane, which results in apoptosis [9].

The surface functionalization of CNMs has been employed to improve their biodegradability, safety, and aqueous solubility. The functionalization of CNMs improves delivery efficiency through reduced clearance and prolonged retention in the body [7]. However, some research has shown that some methods of surface functionalization, such as cationic or anionic functionalization, may lead to higher toxicity compared to non-functionalized CNMs. Evidence has shown that the functionalization of CNMs creates a more dynamic material through the “tagging” of different drugs, peptides, nucleic acid, amino acids, and proteins on the surface of CNMs, which can enhance their efficiency, and solubility while reducing their toxicity [10,11]. Various interactions, such as covalent, ionic, Van der Waal or π–π, can be used to link different functionalizing agents and/or biologically active molecules to the CNMs [11]. These interactions help to graft the functional groups over the surface of CNMs and allow for strategic placement and functionalization at specific regions of the CNMs’ shape [12]. Because these modified CNMs feature various inherent properties, they are extensively used. Therefore, we conducted a review of CNMs, emphasizing the different types of CNM, their importance in drug delivery, and their role in biomedical applications.

## 2. Fabrication of CNMs

**Figure 1:**
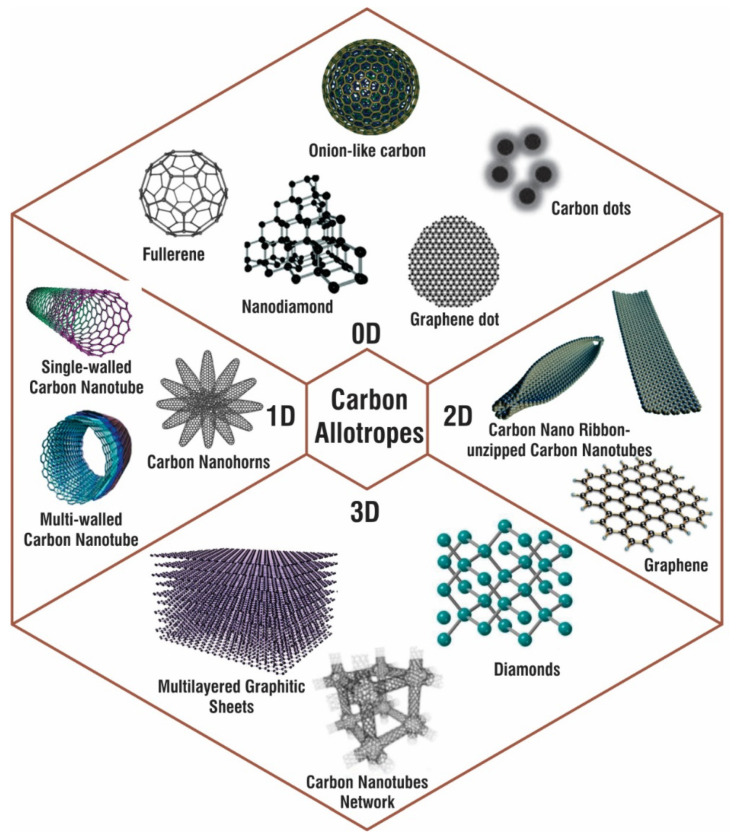
Various nanoforms of carbon allotropes with examples for 0D, 1D, 2D, and 3D carbon nanostructures.

Carbon is one of the most plentiful elements on the planet. It is found in nature as graphite, diamond, and coal in its basic form. Due to their unique hybridization characteristics and their susceptibility to perturbation during synthesis, nanostructured allotrope forms of carbon have been extensively studied over the last two decades, allowing the precise modification of material properties. There are various hybridization states of carbon (Figure 1). The chemical, mechanical, thermal, and electrical characteristics of various allotropic forms are closely connected to their structure and hybridization state, allowing the same material to be used for a variety of applications.

CNMs can be divided into 0D-CNMs (i.e., fullerenes, particulate diamonds, and carbon dots), 1D-CNMs (i.e., CNTs, Carbon Nanofibers (CNFs), and diamond nanorods), 2D-CNMs (i.e., graphene, graphite sheets, and diamond nanoplatelets), and 3D-CNMs. All decreased dimensionalities, including fullerenes, contain CNMs made completely of sp2-bonded graphitic carbon [12,13]. Figure 1 shows various types of carbon nanoallotropes. Table 1 shows various types of carbon nanoallotropes and their commercial application.

### 2.1. Fullerenes

**Figure 2:**
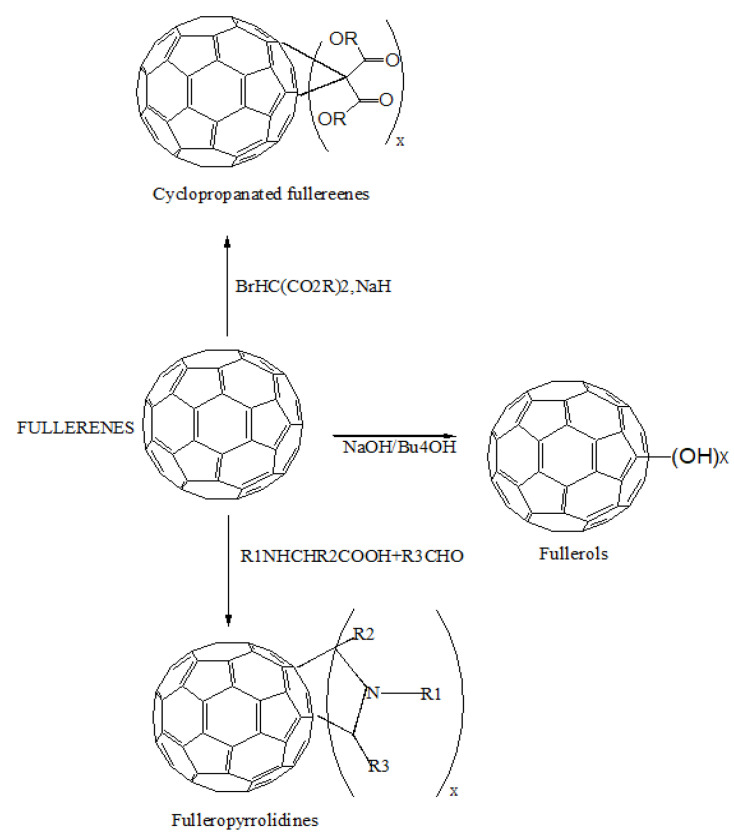

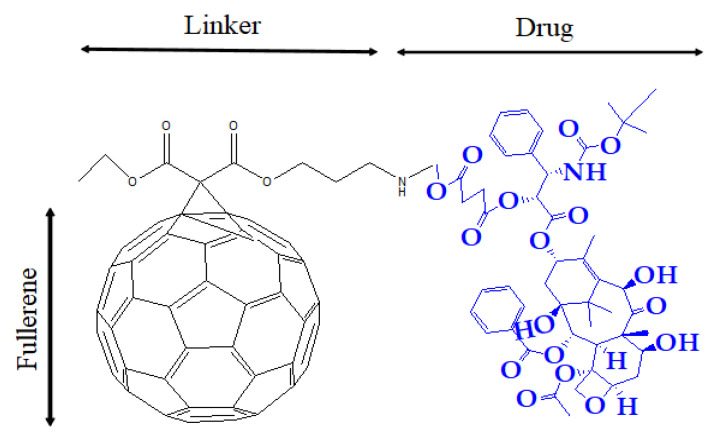
The functionalization of fullerenes and their conjugation with a drug using a linker.

Buckminsterfullerene (C_60_) is the third carbon allotrope, discovered in 1985 by Curl, Kroto, and Smalley. Carbon atoms make up fullerene molecules, which are hollow spheres, ellipsoids, or tubes. The bucky shapes of spherical fullerenes are also known as buckyballs. The enormous curvature of these hollow spheres’ conjugated-electron systems enables rich chemical behavior, permitting the synthesis of a wide range of derivatives, making the fullerene family a useful building block. In medicinal chemistry, it is assumed that three-dimensionality, length, hydrophobicity, and electronic configurations make them an attractive topic. The peculiar configuration of their carbon cage, along with their enormous derivatization spectrum, makes them a possible therapeutic agent. Fullerenes are insoluble in nature; thus, their biological applications have drawn growing interest. The fullerene family, especially C_60_, offer attractive photographic, electrochemical, and physical properties that can be used in several medical fields [27]. Fullerene derivatives have been characterized as inhibitors of human immunodeficiency virus (HIV) [28], contrast agents for magnetic resonance imaging [29], antioxidants [30], and anti-bacterial agents [31]. They are also useful as targeting vectors to mineralized bone [32] and sensitizers for photodynamic therapy [33]. The vast variety of carbon atoms utilized to make fullerenes, the diversified array of moieties that may be hooked up to the surface of fullerene, and the numerous preparation techniques have resulted in a wide range of fullerene derivatives. Among all the types of fullerenes, C_60_ is widely used [34]. Fullerenes’ surfaces are extensively modified to make them water-dispersible, allowing them to be utilized in medicinal and dermatological applications. Of the other fullerene derivatives, C_60_ may be multi-functionalized, forms NP, and serves as a drug absorbent, making it an attractive scaffold for drug administration. Fullerenes can behave as drugs in a variety of functional ways [35]. Fullerenes can produce direct bioactivity, such as antioxidant activity, when surface-functionalized [27]. Both pure and modified fullerenes can penetrate into intracellular space or accumulate at the cell membrane due to their small size (less than 1 nm), posing a threat to cell functions and integrity [36]. In a broad spectrum of applications, pristine fullerenes showed neither acute nor subacute toxicity. However, due to the techniques used for solubilization, the physicochemical characteristics of fullerenes changed, resulting in ROS-related behavior and fullerene toxicity [37]. Figure 2 shows the common approaches, such as Bingel–Hirsch reactions for cyclopropanation, polyhydroxylated for the synthesis of the hydroxyl group with fullerenes, and Prato reactions for the cycloaddition of azomethine ylides to synthesize water-soluble fullerenes. These approaches can increase the solubility of fullerenes to 100 mg/mL [35]. Fullerene derivatives are also helpful in the sustained release of drugs. Fullerene derivatives covalently attached to drugs via a linker were tested for sustained release in lung cancer and were found to be more effective than the naive drug [35].

### 2.2. Nanodiamonds

**Figure 3:**
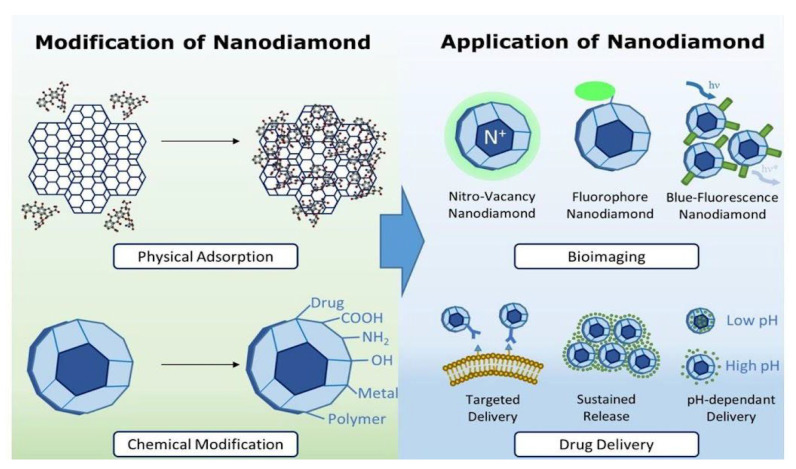
Schematic representation showing the use of combinatorial nanodiamonds in pharmaceutical and biomedical applications [42]. Copyright 2016, Elsevier.

Nanodiamonds (NDs) are carbon nanoparticles that are typically around 2 to 8 nm in diameter, with a truncated octahedral architecture. Generally, the surface of nanodiamonds is coated with a layer of functional groups that stabilizes the particle by reducing dangling bonds. The conversion of sp^3^ hybridized carbon with sp^2^ also results in the increasing stability of the particles. Chains and graphitic patches are formed by the carbons in sp^2^. Oxygen-containing groups end the majority of surface atoms. Some nanodiamonds are faceted, whereas most have a rounded shape [38]. Synthetic diamonds and nanodiamonds are relatively affordable, despite their extremely high cost. Selective uses of nanodiamonds, on the other hand, may necessitate specific physicochemical characteristics, which may necessitate additional surface modifications with various functional groups. The continued study and recognition of the methods of disaggregation, physicochemical characteristics, surface modification, biocompatibility, fluorescence, and optical dispersion of NDs has developed broad perspectives for these biomedical applications [39]. Through their various properties, such as the ability to load high amounts of drugs and the ability to penetrate cellular membranes, NDs have shown tremendous potential to emerge as a medium for transporting drugs into biological systems. The innovative properties of this biomaterial have revolutionized various drug delivery systems and supported gene delivery vehicles in the identification of the biomolecular targets of drugs [40]. Investigation of the biocompatibility, biodistribution, and biological destiny of NDs and their conjugates is required for a therapeutically relevant strategy [41]. Figure 3 displays different methods used in the functionalization of NDs. These methods help to improve the physical properties of the materials and also lead to the acquisition of various other properties, such as targeted delivery, sustained release, and pH-mediated drug delivery. NDs have been widely used for bioimaging because of their excellent optical properties and their various sensing and therapeutic components.

### 2.3. Carbon Quantum Dots

**Figure 4:**
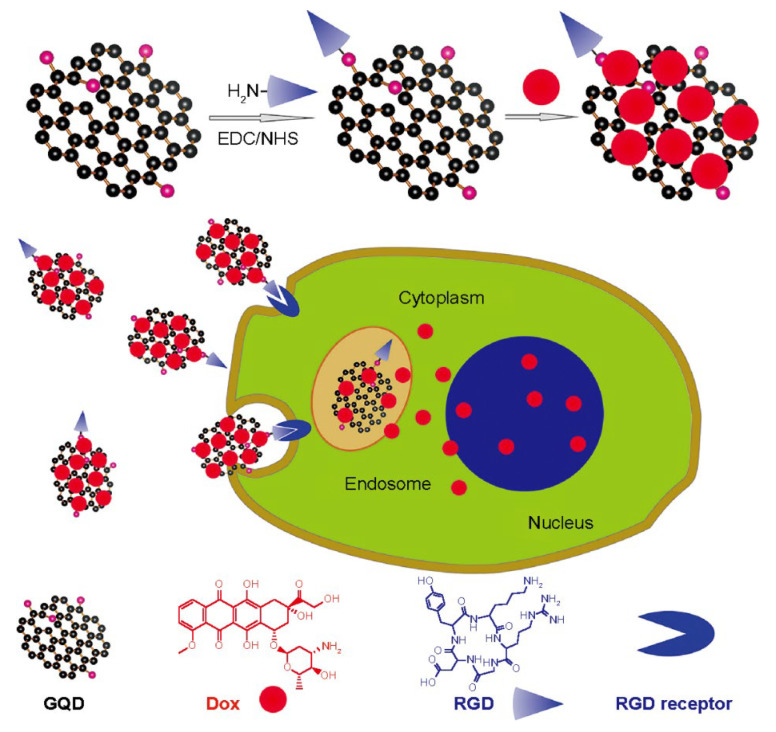
Schematic presentation of cancer cells being treated by multifunctional GDQs [51]. Abbreviations: eDc/Nhs, 1-(3-(dimethylamino) propyl)-3-ethyl carbodiimide and N-hydroxysuccinimide; GQD graphene quantum dot, Dox, doxorubicin; RGD, arginine glycine-aspartic acid. Copyright Dove Press, open access to scientific and medical research.

Carbon quantum dots (CQDs), which include GQDs and CQDs, are a kind of carbon nanoparticle with a size of less than 10 nanometers. CQDs have lately been the focus of research because of their fluorescent, nontoxic, and water-dispersible properties. At high temperature and pressure, CQDs can be made from graphene, cellulose, or other materials. Due to their unique properties, CQDs have received much attention. CQDs are fluorescent carbon nanostructures with two origins of the fluorescent properties, the emission of fluorescence from conjugated π-domain bandgap phases and fluorescence from surface defects. Various elements, such as nitrogen, sulphur, and phosphorus have been used in doping CQDs to improve their properties. They also help in Reactive Oxygen Species (ROS), through the hydroxyl free radical scavenging properties of doped ions [43], and photoluminescence, by increasing their fluorescence brightness and shifting their emission spectra [44,45,46]. From biological imaging to electrical and photonic devices, CQDs bring up new possibilities. Carbon QDs are notable for being constructed out of a plentiful and largely harmless element, which can aid in the ecologically responsible development of solar technology [47]. In the near-infrared (NIR) spectral area, CQDs can emit fluorescence, making them ideal for biomedical applications. CQDs have shown benefits in various sectors, such as biomedicine, solar energy conversion, photocatalysis, light-emitting diodes (LEDs), photosensors, etc. CQDs offer therapeutic uses, including bioimaging, the distribution of drugs, the delivery of DNA, and cancer treatment [48,49]. Figure 4 demonstrates the entry of peptide targeted drug-loaded graphene quantum dots into the cancer cells. These peptides bind with integrins, which are overexpressed in cancer cells. The interior of these cells can be analyzed using fluorescence; it helps in investigating the drug’s release pattern. However, while CQDs do represent an exciting possibility in the drug delivery area, there are still some drawbacks to the technology. These are that the CQDs must be modified for targeted drug delivery and therefore are only as effective as the targeting pathway, the research is quite young, and, although the evidence indicates that CQDs have a low level of toxicity, they are not completely non-toxic. The emerging field does mean that it will take some time to fully characterize and develop an understanding of the long-term effects of CQDs in an in vivo system [50].

### 2.4. Carbon Nanotubes

**Figure 5:**
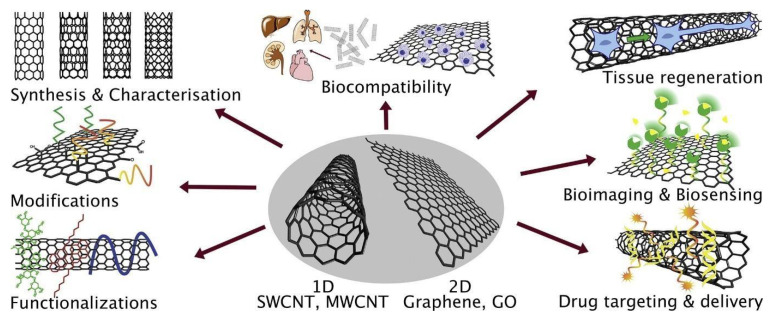
Schematic presentation showing carbon nanotubes alongside their precursor, graphene, and their uses in the biomedical field [63]. Copyright 2018, Elsevier.

In 1993, Iijima and Ichihashi discovered nanometric range carbon, referred to as carbon nanotubes (CNTs) [52,53]. CNTs, commonly known as buckytube, are carbon-based nanoscale hollow tubes. The aspect ratios (length-to-diameter values) of these cylindrical carbon molecules are generally more than 10^3^. These nanotubes are classified as single-walled carbon nanotubes or multiple concentric cylinders, depending on whether they are made up of one tubular nanostructure or several concentric cylinders [54]. Various methods, such as laser ablation, high-pressure carbon disproportion, chemical vapor deposition (CVD), etc., have been used for the formation of these nanocarriers [55]. The CVD method has been the most widely used method due to its high yield capacity. The carbon nanotube obtained using this method has a high degree of length and its morphological characteristics are more enhanced [56]. There are three forms of carbon nanotubes: single-, double-, and multi- walled carbon nanotubes. Single-walled carbon nanotubes (SWCNTs) and double-walled carbon nanotubes (DWCNTs) are made up of one or two (concentric) graphene cylinders, but multi-walled carbon nanotubes (MWCNTs) are made up of many concentric cylindrical shells of graphene sheets. CNTs have poor water solubility; thus, to enhance the solubility rate of CNTS, functionalization approaches are being developed. To functionalize CNTs for increasing their solubility in water, CNTs are covalently or non-covalently functionalized. Bensghaïer et al. used various dyes, such as Azure A (AA-N^2+^), for the surface modification of MWCNTs; the formation of hybrids was useful in different applications, such as biosensors and optically pH-responsive materials [57]. In another research study, biocomposite particles were prepared using lipase and MWNTs to enhance their solubility in solvents: lipase from *Candida rugosa* was covalently anchored onto acid-treated MWNTs through a self-catalytic mechanism [58]. Furthermore, a quantitative assessment of the degree of functionalization is more reliable in general. The benefits of the covalent method, however, are counterbalanced by the adverse disruption of the CNT’s conjugated backbone, which can significantly impair electrical and optical characteristics and, in many cases, limit their performance for certain applications that need such qualities. A quantitative analysis of the degree of functionalization is in general more reliably assessed. Many experiments on the use of surface-functionalized CNTs in the biomedical field have been performed during the past decade [54]. As shown in Figure 5, various medical applications are used in the biological process of carbon nanotubes, such as drug delivery, biosensing, photodynamic therapies, drug discovery, etc. [59,60,61]. 

There is a high degree of interest in the use of carbon nanomaterials in pharmaceuticals. This is due to their highly tunable characteristics and certain desirable traits that they contain inherently. CNTs have very good surface areas and, therefore, an extremely good drug loading capacity, which is naturally a very desirable trait for drug delivery. Along with their nanometric scale and the ability to functionalize them in a variety of ways, their drug delivery capabilities are a growing area of interest. Of course, there are some inherent flaws in current CNTs that hinder their use in pharmaceuticals and will need to be overcome. This is primarily that they do not show good biodegradability and, by extension, may be toxic with long-term or prolonged use [62].

### 2.5. Carbon Nanofibers

CNFs are members of the covalent CNM family and feature conductivity and stability similar to CNTs. The stacking of graphene sheets of various forms distinguishes CNFs from CNTs, resulting in more edge locations on the outside walls of CNFs than those of CNTs. This may make it easier for an electroactive analyte to transfer electrons [64]. CNFs are ideal candidates for next-generation on-chip connection materials, as well as potential immobilization substrates, due to their unique chemical and physical characteristics [65]. Furthermore, when compared to CNTs, CNFs are less costly; thus, they are easily used in the textile industry [66]. CNF is favored for electrical and thermal conductivity because it has a higher degree of crystallographic alignment. It not only has the same low density, high modulus, high strength, high conductivity, and thermal stability as carbon fiber manufactured using a chemical vapor deposition growth process, but it also offers benefits such as a low defect rate, a large aspect ratio, and a huge surface area [67].

**Figure 6:**
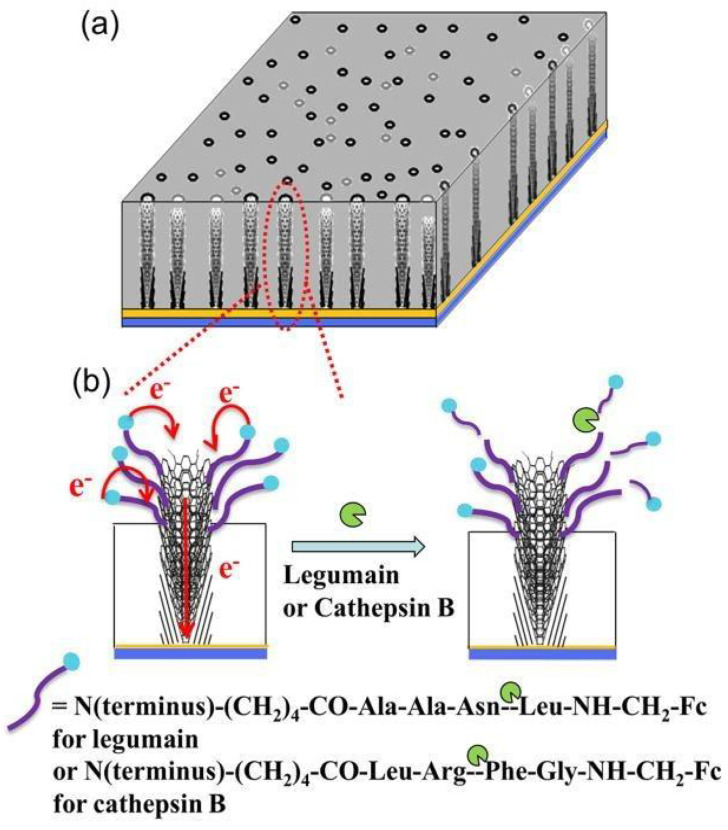
Biosensors formed using carbon nanofibers showing distinct properties. (**a**); represent CNFs vertically aligned in SiO_2_ matrix. (**b**); shows electrons transfer and loss of electrochemical signal from ferrocene attached at the distal end of peptide. This loss of signal is due to the cleavage of peptide at a specific site [70]. Copyright 2013, American Chemical Society.

CNFs are synthesized via chemical vapor deposition, phase separation electrospinning, and templating [20]. Among these, electrospinning has steadily gained popularity as a low-cost and simple method of manufacturing nanofibers [68]. CNFs can be modified for biomedical applications with many different bioactive molecules [69]. Figure 6 demonstrates a nanoelectrode array-based electrochemical technique for detecting protease activity. With a ferrocene (Fc) moiety connected at the starting end, vertically aligned carbon nanofibers were covalently bonded with legumain and cathepsin B. Fc measured the signals and identified different distinct properties from carbon electrodes. 

The carbon backbone and synthesis methods of CNFs allow the application of varied functionalization methods. These methods allow the material to be used for a diverse range of applications. The nanofibers can be modified and functionalized via three primary methods: firstly, through direct incorporation during the electrospinning fabrication with a variety of compounds, such as metal ions, other biomaterials, or active biomolecules and drugs; secondly, the adsorption of relevant molecules to the surface of the CNF’s fibers post-fabrication; and, finally, a combination of both techniques, in which modifications during the electrospinning fabrication are then used as covalent anchor points for surface modifications using relevant chemical pathways.

### 2.6. Graphene Nanosheets

**Figure 7:**
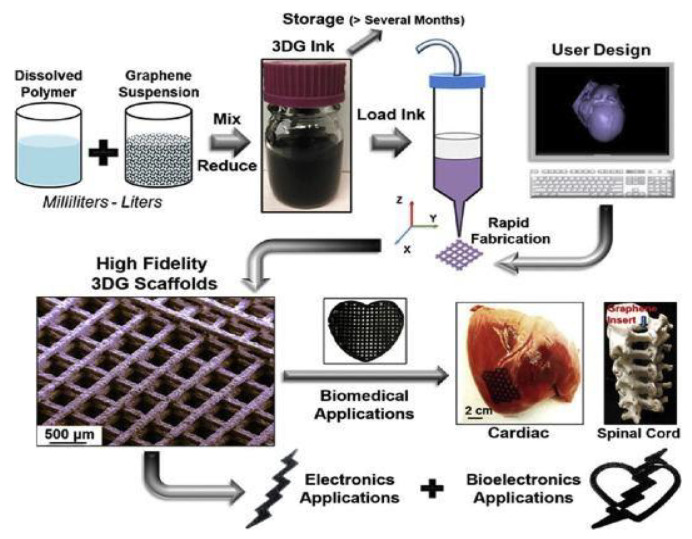
Production of a 3D printable graphene composite scaffold for biomedical application [78]. Copyright 2015, American Chemical Society.

Graphene is an allotrope of carbon, made up of a single sheet of atoms organized in a honeycomb lattice in two dimensions. The name is a combination of "graphite" with the suffix -ene, and it refers to the graphite allotrope of carbon, which is made up of stacked graphene layers. Graphene is a two-dimensional sheet of sp^2^-hybridized carbon atoms organized in a hexagonal lattice [71]. Graphene has gained traction among both the science and technical communities due to its mechanical, electrical, and thermal properties [72]. Its remarkable characteristics, on the other hand, can only be demonstrated experimentally in samples with a high degree of lattice perfection. Structural flaws are inadvertently introduced into the lattice during growth or by physicochemical treatment, which can dramatically impair the graphene’s characteristics and, as a result, the performance of graphene-based devices [73]. Melt intercalation, in situ polymerization, and solution mixing are the most common techniques for incorporating graphene into polymer matrices [74]. Recently, there has been significant progress in the creation of graphene derivatives, such as graphene oxide, porous graphene/graphene oxide, reduced graphene oxide, and GQDs. Another important kind of graphene is the zig-zag pattern found in the structure of graphene commonly called nanoribbon [75]. Various other new forms of graphene have also come into the limelight, such as aerogel graphene, gyroid graphene, etc. [76]. Figure 7 demonstrates the creation of 3D-printable graphene by combining graphene with polylactide-co-glycolide (3DG). Human mesenchymal stem cell (hMSC) adhesion, survival, multiplicity, and neurogenic differentiation have all been aided by 3D printed carbon materials, with the notable overexpression of glial and neuronal genes. Another growing area of research for graphene nanosheets and their derivatives is as a drug release mechanism. Although this application is still in its infancy, there have been initial experiments performed targeting cancerous cells and the controlled release of tegafur [77]. Other applications being investigated are the use of graphene nanosheets and their derivatives as additives in hydrogels to improve their physical characteristics, such as rigidity and conductivity, and their suitability for biological systems, i.e., cell adhesion and proliferation [16]. The current difficulty with graphene and its derivatives is the assurance that during fabrication, single-layered carbon sheets are formed and not sheets of multi-layered graphene. This is essential as the structure and symmetry of the bonds are the key characteristics of the sheet.

## 3. Drug Delivery Systems

**Figure 8:**
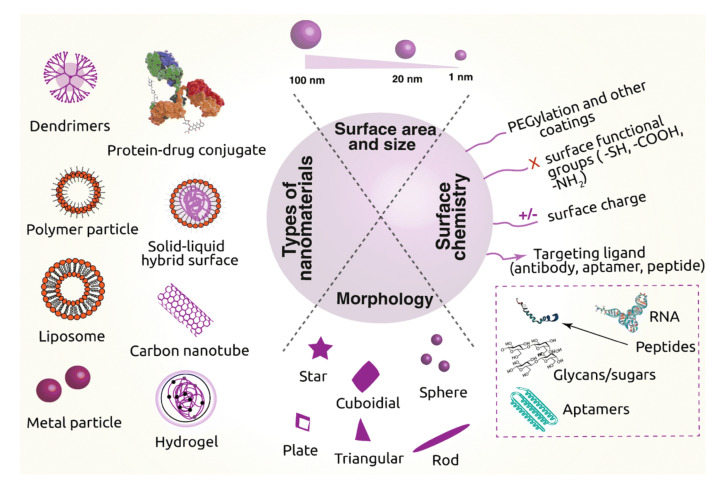
Current trends in drug delivery systems employing nanomaterials as drug carriers [84]. Copyright Springer Nature, 2019. Open access.

Nanomaterials have gained enormous recognition due to their inherent capacity for drug delivery. Many research groups have studied nanomaterials (polymeric nanomaterials, CNMs, inorganic nanomaterials, and nanohybrids) for drug delivery through the loading and controlled release of medicinal products. The use of CNMs in drug carriers minimizes side-effects and reduces both the overall dosage and the dosage frequency. The ability to modulate the size, morphology, and surface functionalization of CNMs make them a useful delivery vehicle for encapsulating and transporting the drug entity to various parts of the body [79,80]. CNMs such as C_60_, GQDs and NDs feature distinct chemical and physical properties or biological effects compared to larger-scale counterparts (graphite), which can be beneficial for drug delivery systems. Some important advantages of CNMs are their small particle size and high surface-area-to-volume ratio, as well as their capacity to bind with drugs/biomolecules to enhance uptake across the plasma membrane. C_60_ and the delivery system based on it are considered very encouraging for antiviral drugs that play a pivotal function in the HIV treatment. The fullerene-based derivatives also prevent HIV protease as a result of constructing a complex, and it was shown that dendrofullerene has the highest anti-protease activity [81]. There are reports that DOX conjugated with C_60_ demonstrates controlled release and positive antitumor activities. Other derivatives, such as fullerenols, have high solubility in polar solvents, which results in greater carrying capacity for several drugs. Thus, they are potentially useful for delivering and improving the efficacy of anticancer drugs [82,83]. Figure 8 displays trends in drug delivery systems using different nanomaterials with their various parameters, such as morphology (spherical, cuboidal, triangular etc.), size (1 nm to 100 nm), surface area, and surface modification through the addition of functional groups (–SH, –COOH, –NH_2_) or the targeting of ligands (as antibodies, peptide, aptamer, RNA) as drug carriers.

Fine-tuning CNMs characteristics for optimal drug delivery requires the consideration of the following characteristics. Nanoparticles’ surface-area-to-volume ratio could be changed to allow more drug molecules to bind [85]. A further key design consideration is the surface functionalization of CNMs, which is routinely performed through bioconjugation or the adsorption of compounds onto the CNM’s surface. Surface-functionalized CNMs with appropriate ligands can improve drug binding, suppress the immune response, or enable the targeting/controlled release of desired molecules. By taking advantage of these features, it is possible to attain increased efficacy and reduced toxicity. More drugs are supplied to the target site, increasing efficacy and limiting the amount of the drug in the body, which reduces hazardous side effects [86]. A surface-modified and DOX-loaded CNT-based novel drug delivery system can deliver an anti-cancer drug to the tumor site with limited interaction with other systems and organs. 

**Figure 9:**
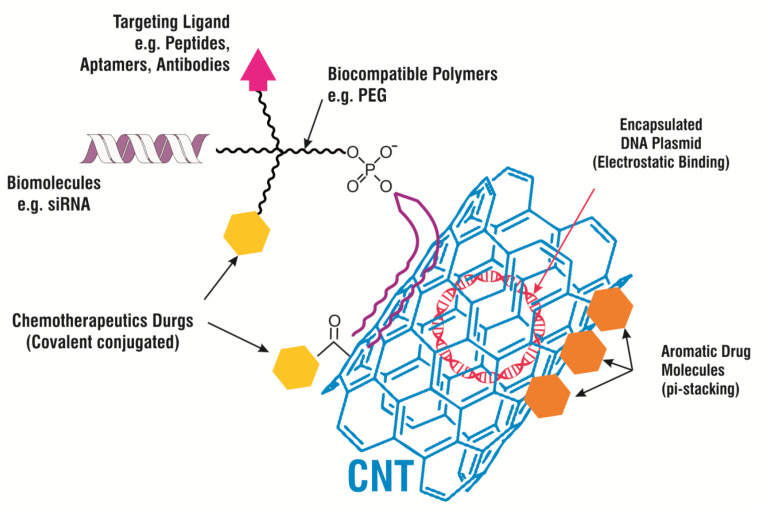
A schematic diagram presenting several strategies for CNT-based drug delivery and cancer therapies.

Until now, countless methods have been developed by many researchers to carry smaller compounds, such as chemotherapeutic anti-cancer drugs, on CNMs, either by using covalent conjugation or non-covalent adsorption. The design of CNM-based drug vehicles has also been led by theoretical modelling. Drugs are covalently conjugated with a nanocarrier and attached to functional groups on the CNM’s surface or coated with polymers on the CNM via cleavable bonds. Sahoo NG et al. [87] used folic acid (FA) to modify the surface of CNTs, which resulted in the enhanced suppression of tumor growth and minimized the side effects caused due to the DOX in the solution. The developed formulation with a modified surface of CNTs and with a high DOX loading capacity boosted anti-tumor efficacy in the in vitro study. This study offered better hope for the development of a promising therapy with lower systemic toxicity that might be utilized for tumor treatments in the future. Similarly, CNMs in conjugation with other anticancer drugs, such as docetaxel [88], tamoxifen [89], and oxaliplatin [90] have also shown encouraging results for the treatment of cancer in vitro, as well as in the in vivo study. Table 2 emphasizes different types of drug-loaded CNMs studied for the assessment of efficacy in vitro and in vivo against several diseases. Pei X et al. [91] demonstrated that anticancer drugs conjugated with PEGylated graphene oxide (GO) can be delivered to cancer cells in the cell culture. This study suggested that a novel delivery system could be used for the delivery of different aromatic, low-solubility drugs, which could support the development of better and more efficient treatments for cancer. In another in vitro study, PEGylated nano-GO loaded graphene oxide with DOX was formulated and showed it can be used as chemotherapy and photothermal therapy (PTT) for the treatment of cancer. In the safety assessment study, this delivery system did not show any cytotoxicity to the murine mammary tumor cell line EMT6 [92,93]. Figure 9 demonstrates several perspectives employing CNT-based drug delivery carrier systems. These approaches include the binding of the ligand at its particular site (such as the antibody, peptide etc) in disease, single-stranded biomolecules (such as siRNA, miRNA) or the coating of biocompatible polymers to provide intimacy in the living system (e.g., Polyethylene Glycol (PEG)), some drugs are chemically attached to the nanocarrier’s surface or PEG (e.g., chemotherapy drugs) and plasmid encapsulated by CNT that could be helpful in detection and imaging [94]. 

## 4. Biomedical Scaffolds

Biomedical scaffolds are engineered materials that are put inside or on the surface of the body. These materials are designed specifically for interacting with the cellular environment, resulting in the formation of new tissues that are beneficial for medical purposes. Cells seeded in these scaffolds support the formation of three-dimensional structural tissues. Many scaffolds or implants deliver medication, monitor body functions, and provide support to organs and tissues. Medical scaffolds are synthetic and can be used by the human body to mimic the normal function of the missing or damaged biological structure [102]. These devices are made up of chemically inert, very strong, fatigue-resistant, cheap, and corrosion-resistant metals and alloys [103]. Metals and their alloys have been used predominantly as structural biomaterials for different applications in reconstructive surgery, mainly orthopedics, and in blood vessels [104]. 

CNMs in specific 1D and 2D CNMs have been used for improving the strength of orthopedic implants. CNTs and graphene nanosheets have been added to bone cement composites due to their enhanced fatigue performance, which can lead to improved longevity of the implant. The SWCNTs and MWCNTs that are used as fillers on the polymethyl methacrylate (PMMA) mixed resin matrix have been found to offer more advantageous physical characteristics, which allow growth and support the adhesion of newly formed tissue throughout the implant [105]. Krul et al., in 2007, demonstrated that nanocomposite implants made up of poly-D, L-lactide, and MWCNTs dispersed slowly in comparison to those composed of a single polymer without additives [106]. Yu Bai et al., in 2016, prepared and characterized a composite implant with reduced GO for medical applications. It was found that GO-based fluorhydroxyapatite implants restrict the adhesion and multiplication of *Streptococcus mutans*. The results show that the composite implant would be invaluable as dental implant material, and for other bacteria-free implants [107].

A novel biocompatible nanocrystalline diamond (NCD) coating technology was used in the generation of CNMs. NCD coating provides unique mechanical, electrical, chemical, tribological, and biocompatibility features, allowing them to be used to fabricate a new range of biomedical scaffolds and tools with higher performance as compared to the existing alternative, as well as materials that have recently been used in commercial devices and implants but whose performances are constrained [108].

The electrical and physical properties of CNMs make them a promising material for implantation where both mechanical and electrical stimulation is required. Platforms designed around these properties can benefit both tissue engineering and transition from in vitro to in vivo applications for implantable applications. Table 3 represents various types of medical implants based on CNMs. In particular, the focus has been on neuronal and peripheral nerve cell applications, as natural healing pathways in this area are limited and the damage is often permanent. Ongoing research aims to provide a means for repair or even circumvention of this damage. Work conducted by Ghosh et al. shows that electrospun nanofiber scaffolds containing MWCNTs demonstrated excellent regeneration properties for peripheral nerve cells in rats [109]. The nerve cells regenerated directionally, an important characteristic for functional nerve regeneration.

Similarly, CNMs can be used for neuronal applications and deep brain stimulation. Deep brain stimulation is a form of electrotherapeutics, in which the brain is stimulated via implanted electrodes to combat conditions such as epilepsy [110]. Conductive applications are required not only for the stimulation of the neuronal cells but also for monitoring the electrical signals of the brain and to anticipate electrical misfiring in the brain to induce the corrective stimulation pulse required. The conductivity and nanoscale of CNMs are ideal for this and development is well underway to take advantage of them for these purposes. Groups such as Alvarez et al. have been demonstrating the remarkable application of these electrodes in a variety of species, with exciting results [111].

## 5. Tissue Engineering

Tissue engineering is a multidimensional science that utilizes the application of biological sciences, medicine, and engineering to regenerate or mimic tissue material for organ transplantation, therapeutic and diagnostic purposes [117,118]. Biomaterials play an essential role in tissue engineering because they can stimulate certain biological processes, modify cell differentiation, and influence cell–cell interactions [119,120]. Hybrid or composite materials are used to adequately mimic the physical and biological characteristics of the original specialized cells, as a single material may not fulfill all the requirements to fabricate artificial tissues [121]. CNTs are inert and non-biodegradable, which makes them suitable for scaffold production for tissue regeneration, as well as favorable surfaces to promote the transmission of neural signals [122]. Mazzatenta et al. found that SWCNT with the hippocampal cells might be directly involved in stimulating the activity of the brain circuit, considering it is a favorable material for tissue engineering. Non-functionalized aligned MWCNTs were found to promote the growth and multiplication of pancreatic cancer cells, demonstrating a new way to analyze cancer in vitro [123]. Abarrategi et al. reported the development of CNT/chitosan meshes that supported cell recolonization and also noticed their break up in vivo through proper dispersion in the newly raised tissue [124]. CNTs resemble certain biological structures; therefore, they are used as mimicking agents. The morphological resemblance of CNTs to fibrillar/extracellular matrix protein and their capacity to promote cell adhesion makes them suitable for use as synthetic substrates for artificial bone formation.

Graphene was demonstrated to have a unique ability to adsorb nucleobases via π–π interaction and also to effectively protect nucleotides from enzymatic degradation [125]. Graphene nanosheets could be used as a suitable vector due to their easy uptake by cells. Chen et al. developed a poly (ethylenimine)-GO (PEI-GO) as a transfecting agent to deliver plasmids into HeLa cells and found that the PEI-GO enhanced the transfection efficiency by due to a proton-sponge effect [126]. In the application of tissue engineering, graphene and its derivatives could be bound with other biomaterials to improve their mechanical, physical, and electrical characteristics. The modification of the surface attributes of graphene with a coating of SiO_2_ enhances the proliferation and stretching of human mesenchymal stem cells [127]. Artificial fibrous structures that permit a greater transportation of nutrients are broadly employed in biomedical applications. Graphene-based fibers report higher conductivity as compared to composite fibers [78]. A biocompatible GO scaffold increased cell proliferation and HFB4 cell attachments to a fibrous composition. It also helped in enhancing mechanical properties and allowed manipulation at the nanoscale level in fibrous scaffolds for biomedical applications [128]. Table 4 shows use of CNMs in tissue engineering application.

Nanohydroxyapatite/graphene oxide (nHA/GO) composites, which increase cell survival, were successfully modified by polyethylene glycol, polyvinylpyrrolidone, and chitosan [129]. A nHA/GO composite modified with synthetic polymers improved the growth, viability, and proliferation of MG-63 cells for more than 14 days. The modified nHA/GO composites, which are limited when applied in traditionally-used composites of natural polymers, showed enhanced bioactivity [130].

## 6. Wound Healing

**Figure 10:**
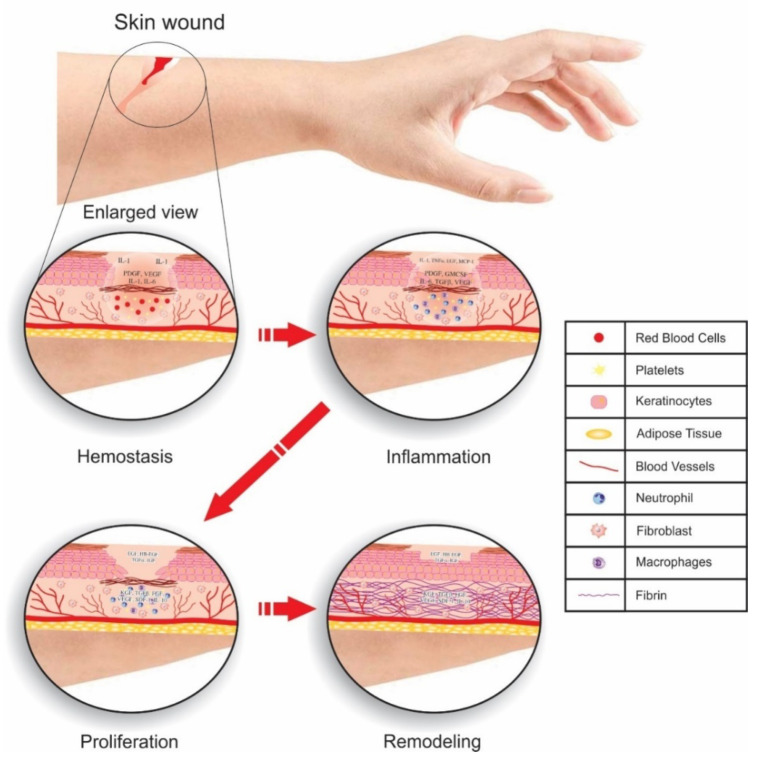
Distinct phases of wound healing in skin illustrate cells and molecules in the tightly controlled process of recovering a healthy barrier.

Wound healing is a distinct physiological process through which injured tissue is repaired within a short time. Uncoordinated wound healing is generally associated with many health problems, such as diabetes, extensive burns, and chronic wounds [137]. In wound healing, there are four coordinated and overlapping phases [138]. These phases must proceed in the correct sequence for proper healing [139]. Figure 10 indicates 4 evolutionarily conserved phases of wound healing in skin executed in a coordinated manner: hemostasis, inflammation, proliferation, and tissue remodeling. Multiple cell types (fibroblast, keratinocyte, endothelial cells etc.) and molecular events are involved in highly regulated ways by various growth factors, cytokines and chemokines [140].

Researchers have used graphene with ultrafine silver nanoparticles to demonstrate the antimicrobial and burn wound healing potential of the material. Tuning the formulations of the materials generated a large number of oxidative radicals that lead to the development bactericidal properties. Histopathological studies revealed that graphene-based nanomaterials can enhance the regeneration of the epidermis, thus demonstrating their promising application to burn and wound healing [141].

Silver nanoparticle (AgNPs)-guided single-stranded DNA (ssDNA) attached to graphene oxide (ssDNA-AgNPs-GO) exhibit good bactericidal activity as well as wound healing properties. This antibacterial activity has been observed through synergistic antimicrobial properties used against *Escherichia coli*, *Pseudomonas aeruginosa*, *Staphylococcus aureus,* and *Bacillus subtilis* at very low minimum inhibitory concentrations (MIC). Due to its improved antibacterial and wound healing properties, ssDNA-AgNPs@GO offers broad applications against bacterial infections caused at the sites of damaged tissues [142].

Fullerenes and CNTs accelerate wound healing by reversing the inflammatory and proliferative phases. Fullerenes, which are stronger antioxidants than CNTs, target and inhibit ROS and reactive nitrogen species generated at the site of damaged tissue [104]. Researchers have demonstrated that fullerene imitated accelerated wound healing in a modified scratch assay and an ex vivo human skin assay. CNMs have been used to promote cell migration, induce wound closure in human skin explants, and increase the speed of wound healing. Consequently, fullerene derivatives are promising wound healing agents that may assist the development of better treatment designs [143]. Table 5 represent different types of CNMs in wound healing application.

## 7. Biosensors

**Figure 11:**
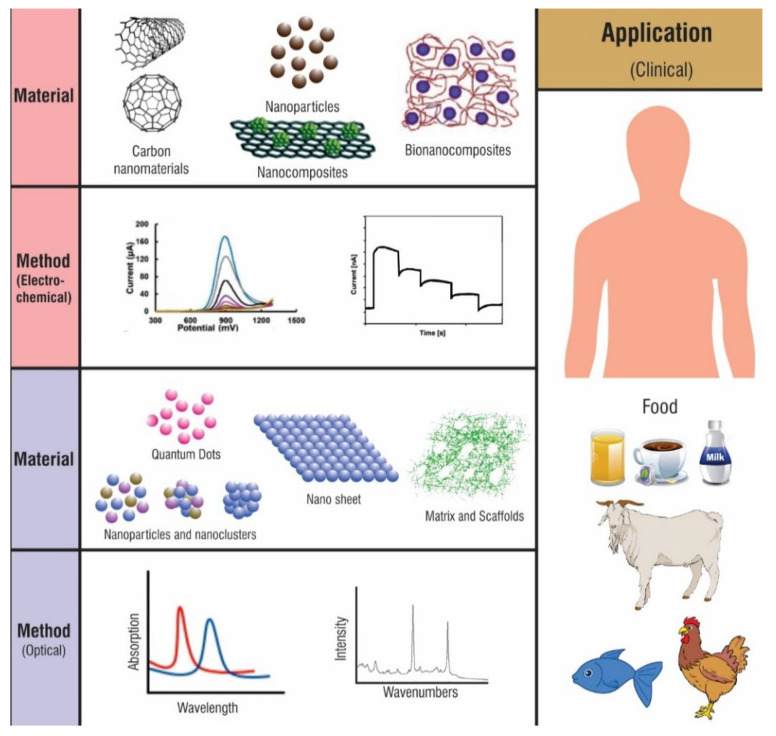
Schematic representation of the possible application of CNMs implemented in biosensing applications.

Biosensors are sensors whose analyte or recognition elements are of a biological nature. Thus, they can be used to track changes in biorecognition events in a diseased or abnormal condition [150]. In general, a biosensor is an element comprising a transducer producing a thermal, electrical, or optical output signal. In electrochemical biosensors, carbon materials have been used for decades [151,152]. Figure 11 shows different types of CNMs, of which two types of methods, electrochemical and optical, are most commonly used for biosensing platforms. In the electrochemical sensing platform, CNMs improved analytical performance due to the increase in electrochemically active surface area. In optical sensing platforms, CNMs are employed as fluorescence emitters and fluorescence quenchers due to changes in fluorescence intensity. CNMs offer a diverse set of uses in the clinical, agricultural, and food industries because of their simpler, portable, and less expensive disposable biosensors [153].

A broad range of ions and compounds, such as glucose, fluoride ion, hydrogen peroxide, and other organic vapors, have been detected using functionalized fullerenes and modified electrodes carrying fullerenes [154]. To be used as a sensor, Carbon Nanodiamond (CND) needs surface functionalization to enhance its solubility and facilitate specific binding to the analytes of interest. CNDs can form emissive nitrogen-vacancy (N-V) defects where the fluorescence depends on the N-V center’s electronic spin state showing unique properties of CNDs [155,156]. In recent years, fluorescent CDs have gained a foothold as fluorescent probes for the identification of several cations, such as Cu^2+^, Zn^2+^, Al^3+^, Ag^+^, K^+^, Be^2+^, Hg^2+^, and Fe^3+^, as well as, numbers of anions, including iodides, hypochlorous acid (HClO), nitrite, oxalate, and superoxide. A number of drugs and small molecules, including tetracyclines, melamine, hydrogen peroxide, 2,4-dinitrophenol, picric acid, amoxicillin, and pentachlorophenol, have been reported by Carbon dot systems [157].

A large number of possible uses for biosensors have been exploited to develop the special physical and chemical characteristics of graphene and CNTs. Graphene and CNTs can be altered with a biological sensing component in these biosensors, such as nucleic acids, and can also be changed with suitable groups capable of biomolecular detection (e.g., proteins, such as enzymes and antibodies) or bioprocess tracking. Biosensor designs are also influenced by whether the investigation is conducted in vivo or in vitro. In many significant reviews that have reported the development of graphene and CNT biosensors, these factors have been discussed [158,159,160]. Many researchers have designed CNT-based glucose sensors that can detect glucose levels in biological samples based on glucose oxidase-impregnated polyvinyl alcohol solutions [161,162,163,164]. Researchers designed an electrochemical biosensor for glucose oxidase sensing that was helpful in studying the structure of glucose-oxidase-coated MWCNT [165]. Table 6 represent various application of CNMs in biosensing application. Nitric oxide and epinephrine can be also detected using CNT-based electrochemical biosensors [166,167]. Graphene was also used to fabricate an enzyme substrate based on an ultra-efficient probe for the sensing applications of the different biomolecules [168,169]. Researchers have also used fullerenes as materials for sensors and biosensors in the detection of deoxyribonucleic acid (DNA) [170]. The immobilized DNA was used in a fullerene-infused screen-printed electrode for the detection of 16S rDNA. The efficacy of the established procedure was examined by spotting 46S rDNA of E. coli on the fullerene-impregnated electrode with probes that were ideally aligned [171].

Carbon nanohorns (CNHs) offer an appealing alternative to CNT-based sensors [172]. Other unique properties of CNHs, such as their electrocatalytic activity against the oxidation of dihydroxybenzenes, can be used as methods of detection; for instance, the identification of food pollutants, such as bisphenol A, malachite grey, and triclosan [173,174]. CNH-modified standard glassy carbon electrodes have also been employed for the rapid identification of isomers of dihydroxybenzene catechol, hydroquinone, and resorcinol, with limits of detection of 0.1 M, 0.2 M, and 0.5 M, respectively [175]. CNH optical-based sensors typically depend on the ability of the CNH to quench an analyte’s fluorescence in the absence of intrinsic fluorescence. This technique was reported by Zhu et al. for the detection of DNA. A mix-and-detect technique for the fluorescent detection of an HIV-related oligonucleotide sequence has been demonstrated [176,177].

## 8. Bioimaging Applications

Bioimaging is a noninvasive imaging technique that can be used to visualize living organisms, organs, cells, or internal cell structures, based on their biological activity. It aids in the study of the function of a particular organ in different conditions in correlation with the anatomical 3D structure of specimens. These imaging techniques do not interfere with organ functions, such as respiration, movement, etc. Through the use of high-resolution imaging platforms such as Stimulated Emission Depletion (STED) microscopy, they can also facilitate observations of subcellular structures and all the tissues in multicellular organisms [184]. Bioimaging involves two steps: acquiring and processing images, and picturing the structural or functional aspects of live objects or systems. CNMs are suitable for in vivo and in vitro biological imaging because of their intrinsic optical features, which include a broad absorption spectrum in the visible and near-infrared (NIR) regions, photoluminescence in the NIR range, and significant resonance Raman scattering.

Along with their inherent physical features, particularly their optical attributes, CNMs could be used as biosensors as well as in bioimaging. GQDs, CNTs, and their derivatives possess useful optical features for bioimaging, particularly in living cells, such as visible and NIR photoluminescence, distinctive Raman bands, and photoacoustic and photothermal responses. CNMs have a high ability for fluorescence and the multimodal bioimaging of cells and tissues because of their excellent aqueous solubility, biocompatibility, and minimal cytotoxicity, and even their remarkable tolerance of photobleaching. Intracellular motor protein tagged with SWCNTs has been used to track the dynamic processes within the cytoskeleton. The kinesin-1 motor Ki5c of COS-7 cells was covalently linked to SWCNTs wrapped with DNA. The SWCNT-labelled kinesin motor protein facilitated the real-time tracking of intercellular dynamic events, such as kinesin transport along microtubules and fluctuations in the microtubule-network, using fluorescent microscopy. A complex of SWCNT—a fluorescent, aqueous soluble—and a cytocompatible polymer has been applied for bioimaging. The supramolecular assembly comprises an alkylated polymer that is coupled with neutral hydroxylated or charged sulfated dendronized perylene bisimides (PBIs) and SWCNTs as a stationary base. The backbone of the polymer imparts several new characteristics to the SWCNTs, such as increasing their solubility, making them fluorescent by adding PBIs, and improving cytocompatibility by wrapping around the SWCNT scaffold. In photophysical characterizations and biological in vitro studies, sulphated complexes show better cellular uptake, optical properties, and intracellular staining compared to their hydroxylated analogs [185,186]. Table 7 represented bioimaging application of CNMs.

A suspension of SWCNTs and bovine serum albumin (BSA) was used for in vivo imaging of Drosophila melanogaster larvae. Protein-coated SWCNT was fed to the larvae. Fluorescent images of the digestive system clearly showed peristaltic movements. Besides, the CNDs were covalently functionalized with transferrin protein through carbodiimide and amine functional groups. The transferrin-functionalized nanodots were internalized into cancerous HeLa cells by overexpressed transferrin receptors on the cell membranes and imaged under fluorescent microscopy [187,188].

K. Yang et al. reported labeling of PEGylated GO with NIR fluorophores (e.g., Cy7) and fluorescein isothiocyanate for in vivo and in vitro imaging [189]. Furthermore, in vivo biodistribution of PEGylated GO along with the signal of ^125^I was evaluated by F. Yang et al. [190]. A novel protein-based GO was developed for use in ultrasonic dual-modality for imaging, and photothermal therapy in cancer [191]. Additionally, reduced GO nanocomposites tagged with quantum dots were shown to have implications for PTT, tumor imaging, and in situ monitoring of tumor treatment [192].

The photoluminescent (PL) characterization of nGOs showed that upon excitation, peak PL emission intensity is in the bluish-green region (455 nm). In one study, nGOs encapsulated in polylactic acid (PLA) were shown to be biocompatible and photoluminescent, allowing them to be employed in bioimaging applications [193]. Mesoporous silica nanoparticle (MSN) coated with fluorescent fullerene (C_60_-TEG-COOH) is water-soluble and biocompatible, and it is successfully used for the fabrication of nanocarriers, which have demonstrated a capacity for pH-sensitive drug release and can be put to use for fluorescent cell imaging. In an in vitro study, it was shown that the synthesized materials exhibited excellent biocompatibility. Furthermore, the DOX-loaded nanocarrier showed effective anticancer activity. This study demonstrated a straightforward way to design a dual-purpose nanomaterial, such as for pH-responsive drug delivery, as well as a bioimaging system that can be used as a therapeutic agent and for monitoring treatment responses [194].

## 9. Vaccination

One of the most popular strategies for preventing and reducing infectious and non-infectious diseases is vaccination. CNMs with appropriate surface functionalization can alter the way therapeutic molecules interact with target cells or tissues. In vaccinology, nanoparticles-based formulations enhance immunogenicity by improving the stability, slow-release and cell-targeted delivery of an antigen. CNMs play a critical role in vaccine delivery, since several types of CNMs have been extensively utilized [201].

**Figure 12:**
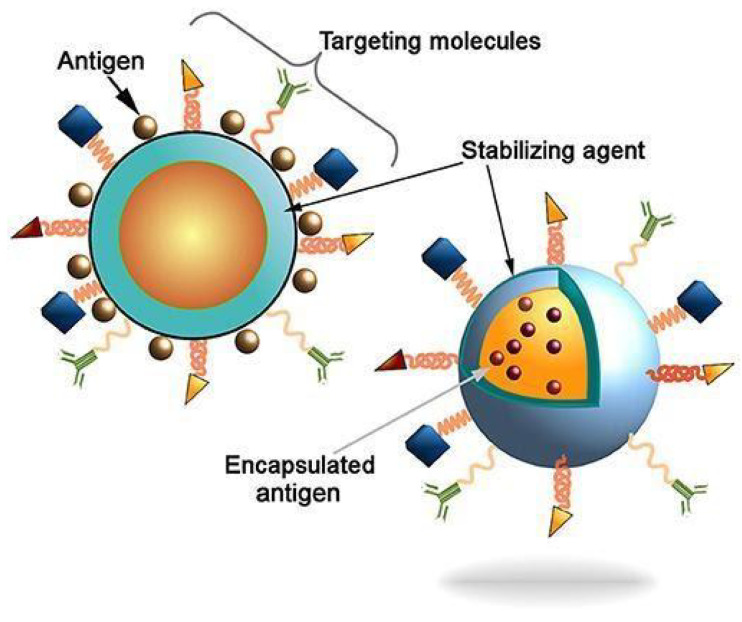
Schematic depiction of antigen delivery through nanocarriers with surface modification to enhance efficacy of vaccine against specific diseases [207]. Copyright © 2021 Pati, Shevtsov and Sonawane. This is an open-access article distributed under the terms of the Creative Commons Attribution License (CC BY).

CNTs offer advantages to the design of a vaccine delivery system with different approaches against viral, bacterial and protozoal disease as antigens as well as CpG adjuvants [202,203,204]. Early attempts to use CNT scaffolds as vaccine delivery vehicles include the covalent attachment of viral envelope peptides of foot-and-mouth disease to CNTs [205]. In this study, it was demonstrated that epitope structures retain their immunogenic nature when linked with CNT. In the animal model, the CNT-viral protein molecular complexes were effective in eliciting particular immune responses against the viral proteins. Pantarotto et al. showed that the peptide-CNT conjugates elicited IgG responses that were specifically able to neutralize antibodies [206]. Figure 12 represents the antigen that might be connected to the surface of the nanoparticles or could be encapsulated. The renovation of the surface of nanoparticles with targeting molecules (e.g., antibodies, fab-fragments, peptides, etc.) potentially improves particle distribution to antigen-presenting cells (APCs) in order to induce innate and adaptive immune responses.

Meng et al. conjugated cell lysate to SWCNTs to study vaccine protection using a murine hepatoma model [208]. The SWCNT-conjugated lysate vaccine enhanced protection levels, as compared to cell lysates only, through the improved activation of cytolytic T cells. Purified protein derivative (PPD) in Freund’s adjuvant generated a predominantly Th-2 response when it was given to the mouse models while, conjugated with an SWCNT, the PPD response was shifted in the direction of a Th-1 [209]. Some researchers studied when the MWCNTs and embryonic stem cells injected into mice suppressed the growth of murine colon carcinoma and enhanced the activation CD4 and CD8 cells. In one study, Villa et al. reported that CNT-peptide constructs might enhance the immunogenicity of a less immunogenic, clinically appropriate cancer-associated peptide [210]. They utilized different methods to conjugate 19-amino acid peptides on SWCNTs, conjugated peptides with an easy uptake by dendritic cells, and macrophages in vitro, and the mice immunized with it demonstrated peptide-specific IgG responses. Some researchers have designed self-assembled fullerenol for dual DNA vaccine delivery for HIV. The conjugate showed promising activity by reducing the antigen dosage and enhanced the protection level, which helps in the activation of signaling pathways [211].

GO is widely utilized for the delivery of biomolecules. It has shown promising results in encapsulating and delivering antigens, as well as the ability to induce the immune system. The functionalization of GOs with hydrophilic groups improve their bio-solubility and biocompatibility; as delivery vehicles, the consequent hybrid GOs possess superior adjuvant properties [212].

Due to the excellent adsorbing properties of GOx, it was used in combination with protein. Consequently, it showed intracellular protein delivery, thus demonstrating its potential use in vaccination. The researcher demonstrated that adsorbed proteins on GO were selectively and effectively taken by dendritic cells and induced the presentation of antigen specifically to the cytotoxic T cells. In this way, it induced a powerful cellular antigen-specific immune response against intracellular pathogens and cancer [213]. Table 8 exhibits recently used CNMs in vaccine implementation.

## 10. Photodynamic Therapy (PDT)

**Figure 13:**
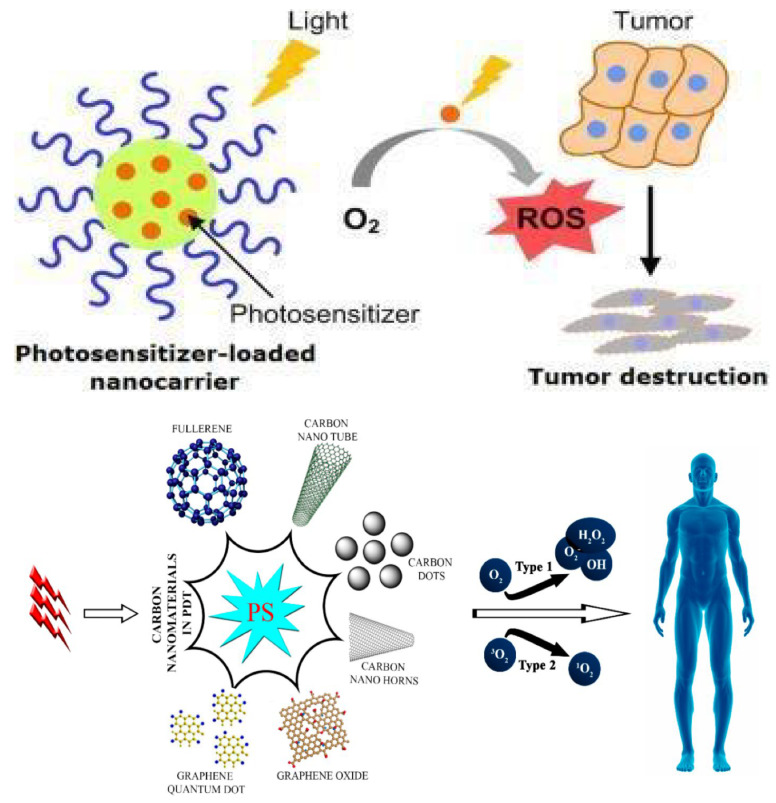
Schematic illustration of the theranostic of disease photo-triggered by carbon-based CNMs, which are targeted and effective in PDT for cancer [223]. Copyright 2016, MDPI, Open Access.

Photodynamic therapy (PDT) is an authentic and easy procedure commonly used in the treatment of cancer. PDT is composed of a combination of light, a class of drugs known as photosensitizers (PS), and molecular oxygen, in order to achieve a therapeutic effect on the target area. Broadly speaking, the therapeutic procedure involves the topical or intravenous administration of a photosensitizing agent to the patient, followed by irradiation with light of a particular wavelength that is within the absorbance range of the sensitizer [220]. Energy from the electronically excited PS is transferred to the ground state of molecular oxygen and generates excited singlet oxygen that is toxic to the cells, causing the death of cancerous cells by apoptosis or necrosis. It also causes serious damage to the cancerous microvasculature, and dramatic changes in the tumor’s surroundings [221,222]. Figure 13 represents the diverse PDT approaches of CNMs: (a) a nanocarrier loaded with PSs sensitizes in the presence of light and converts O_2_ to ROS, which helps in tumor destruction at that specific site; and (b) various types of CNMs are used in PDT applications as shown in Table 9 and after light eradication, PSs in cells produce ROS. The two types of photosensitized processes, Type 1 and Type 2, are based on the type of oxygen consumption.

Ogbodu et al. showed the synthesis of spermidine adsorption on SWCNTS. The conjugation of spermidine, a poly-amine compound, with zinc mono-carboxy phenoxy phthalocyanine results in improving the effect of PDT. The experiment revealed that the cell viability of breast cancer cells lines decreased by 97% [224]. Shine et al. performed an experiment that showed various applications of fullerenes, including the role of PDT and magnetic targeting. The experiment also showed no toxicity of fullerenes during in vivo analysis [225]. For tumor imaging, researchers have developed carboxyl group functionalized fullerene carriers, which can penetrate into cells and can kill cancer cells [226]. Another study revealed the anti-tumor effect and MRI tumor imaging obtained by using fullerenes. The researched conjugated PEG with fullerenes along with pentetic acid, using gadolinium acetate solution. The complex showed an antitumor effect when administered through the IV route [227,228]. Table 10 shows list of patents describe CNTs used in the delivery of therapeutics, imaging and in disease diagnosis.

## 11. Conclusions

Over the past decades, carbon nanomaterials have shown great promise in the development of smart nanomaterials for biomedical applications in drug delivery, bioimaging, biosensing, and tissue engineering. Their uniqueness is due to their increased surface area, substantial mechanical strength, and atypical optical properties; these attributes may prove very beneficial for many biomedical applications. The inherent traits of CNMs, including their carbon-rich nanostructures, tiny size, ease of surface functionalization, and high purity, are key to keeping CNM-based complexes at the forefront of in vivo based studies. However, some limitations might still impede their use in certain biomedical applications in particular drug delivery. Current research focuses on improving the surface chemistry of carbon nanomaterials towards increased bioavailability and biocompatibility and reducing toxicity. Already, researchers have been able to overcome the cytotoxicity of some types of carbon nanomaterials in order to use them as nanocarriers for drugs and genes, as contrasting agents in imaging, as well as scaffolds for tissue regeneration, and various biomedical applications, with exciting results. In our opinion, more research needs to focus on the biomedical applications of CNMs. This would provide researchers in chemistry and biology with an opportunity to overcome the limitations imposed on CNMs by their toxicity and dispersibility. Surface modifications through conjugation and the grafting of more compatible materials would improve CNMs’ toxicity and solubility, which would in turn aid in the development of more biocompatible and soluble materials based on CNMs. We believe that the use of composite materials based on CNMs will expand as their limitations are overcome and they become more appropriate for use in human applications.

## Figures and Tables

**Table 1:** Various forms of carbons and their commercial and laboratory applications [14].

Carbon Based Materials	Presence in Environment and Popular Synthesis Method	Properties	Applications	Ref.
Carbon Nanotubes	Laboratory-scale synthesis Arc discharge, Laser ablation, Chemical Vapor Deposition	High strength, Electronic properties	Biosensors, nanocomposite materials as scaffolds for tissue engineering.	[15,16]
Fullerenes	Manufactured at large scale in industry and laboratory. Chemical Vapor Deposition.	High strength, insoluble in water. Exhibit pi bonding between atom and are stable structure.	Pharmaceutical industry. Found to be beneficial in IT devices and diagnostic purposes.	[17]
Carbon Nanofibers	Laboratory production, chemical vapor deposition, phase separation electrospinning, and templatin.	The thermal conductivity of the molecules is high; they also exhibit greater strength	Cancer therapy, biosensing, tissue engineering, and wound dressing.	[18,19,20]
Diamond	Can be obtained naturally or by artificial means. Rapid pressurisation, pulsed laser ablation	Hard, non-volatile substance.	Used as lubricant in higher temperature. Used in jewellery design, biomedical etc.	[21,22,23]
Graphene	Obtained by artificial means through laboratory production Arc discharge, chemical vapor deposition, mechanical exfoliation	Most reactive form of carbon. Flammable.	Biosensing, bioimaging, bone implantation, drug delivery.	[24,25]
Graphite	Laboratory and industrial production, can be obtained through natural process.	Lubricity, anisotropic, electronics conductivity.	Mechanical heart valves, electrode components, lubricants.	[26]

**Table 2:** Some drug delivery systems based on CNMs with efficacy studies, with specific drugs and target diseases.

Serial Number	Drug Carriers	Drug	Target Disease	Ref.
1	Carbon nanotubes	Metformin	Diabetes	[95]
2	Fullerenes	Paclitaxel Tamoxifen	Lung Cancer Breast cancer	[96,97]
3	Multiwall carbon nanotubes	Diltiazem hydrochloride	Angina Pectoris	[98]
4	Carbon nanotubes	Doxorubicin	Cervical carcinoma	[99]
5	Graphene oxide	Paclitaxel	Lung Cancer	[100]
6	Diamonds	Doxorubicin	Breast Cancer	[101]

**Table 3:** Different medical implants based on CNMs and their biomedical applications.

Serial Number	Carbon Nanomaterial	Medical Scaffolds	Applications	Ref.
1	Carbon nanotube (CNTs)	Hydroxyapatite based CNTs composite.	Helpful in forming a strong bone–implant interface.	[112]
2	SWCNTs	Electrospun polyurethane carbon nanotube scaffolds.	Helpful in differentiation of myoblast cells.	[113]
3	MWCNTs	Polymethyl-methacrylate (PMMA) microspheres, and polyacrylonitrile-based MWCNT scaffolds.	Bone regeneration.	[114]
4	Carbon nanofibers	Collagen-carbon nanofiber scaffold.	Myocardial infarction.	[115]
5	Graphene	Electrosynthesis of polypyrrole (PPy) coating on graphene oxide (GO) nanocomposite.	Improved surface protection and biocompatibility performance in in vitro studies on MG-63 human osteoblast cells.	[116]

**Table 4:** Different uses of CNMs in tissue engineering applications.

Serial Number	Carbon Nanomaterial	Formulation	Tissue Engineering Applications	Ref.
1	Carbon nanotubes	Hydrazide-functionalized carbon nanotubes–pericardial matrix derived from hydrogel.	Improved cardiac tissue engineering.	[131]
2	Fullerene whisker scaffolds	Highly aligned 1D scaffold regulates cellular differentiation to muscle cells.	Promotion of myoblast differentiation to myotube.	[132]
3	Nanodiamond	Poly(l-lactic acid) and octadecylamine-functionalized nanodiamond.	As components of bone scaffolds and surgical tools in regenerative medicine.	[133]
4	Carbon dots	CDs based composite nanofibrous mats.	Guided cell growth and enhancement of cellular activities.	[134]
5	Carbon nanofibers	Electroactive CNF/gelatin (Gel) nanofibrous cardiac patches.	Improved cellular adhesion and proliferation, as well as increased gene expressions and angiogenesis.	[135]
6	Graphene nanosheets	Biomimetic gelatin and bioactive glass scaffolds.	Excellent biocompatibility and engineered stiffness.	[136]

**Table 5:** Different uses of CNMs in wound healing applications.

Serial Number	Carbon Nanomaterial	Wound Healing Agent	Applications	Ref.
1	MWCNTs conjugated with glucose oxidase	Glucose oxidase shows potent antimicrobial activity.	Wound cover or tissue healing matrices.	[144]
2	Fullerenes modified with amino group (C_70_–(EDA)_8_)	Amino groups interact with outer boundary of multidrug-resistant *E. coli* and C_70_ establish a potent hydrophobic interaction with bacteria, which causes cytoplast leakage.	Promising for clinical care of wound infection.	[145]
3	Fluorescent CDs loaded nanocomposites chitosan film	Chitosan, for making film and CDs as crosslinkers are taken, which are biocompatible and used in wound healing management.	Successful formulation regulates the water absorption behavior of chitosan-based film.	[146]
4	Oxygenated nanodiamonds (O-NDs)	O-NDs mimic peroxidase enzymein a rodent model.	Inhibiting and improving the course of periodontal inflammation.	[147]
5	Combination of oral antidiabetic agents-loaded nanofibrous scaffolds	Metformin, pioglitazone, and glibenclamide.	Improved diabetic wound healing on type-1 diabetic rats.	[148]
6	3D graphene foam (GF) scaffold loaded with bone-marrow-derived mesenchymal stem cells (MSCs)	GFs loaded with MSCs clearly facilitated wound closure in animal model.	Enhanced skin wound healing.	[149]

*Escherichia coli* = *E. coli*, EDA = ethylenediamine.

**Table 6:** Different uses of CNMs in biosensing applications.

Serial Number	Carbon Nanomaterial	Biosensors	Targeted Analyst	Ref.
1	SWCNTs	Conjugated aptamer-anchor polynucleotide sequence to near-infrared emissive.	Estimating protein efflux from single organisms in real-time.	[178]
2	MWCNTs deposited between electrodes	CNT resistors.	Detection of Arginase 1 (ARG-1).	[179]
3	Ag-Pt bimetallic electrospunnanoporous CNFs	Modified carbon electrode for dopamine detection.	Dopamine selectively detected in presence of uric acid and ascorbic acid.	[180]
4	Carboxyl functionalized GO (CFGR-COOH)	HRP labelled CFGR-COOH modified with Glassy carbon electrode.	DNA was successfully detected using DPV with ranges between 1 × 10^−6^ and 1 × 10^−14^.	[181]
5	Graphene-bismuth nanocomposite film modified electrode	Immobilized glucose oxidase on nanocomposite.	Successful detection of glucose with good stability and repeatability.	[182]
6	Fullerene (C_60_)	C_60_ acts as donor probe and urea (if present) reacts to DMG and formed DIK acts as receptor on RRS-ET analytical platform.	Successfully developed to detect trace amounts of urea in food.	[183]

(DPV = Differential pulse voltammetry, DMG = Dimethylglyoxime, DIK = 4,5-Dimethyl-2-imidazolone, RRS-ET = Resonance Rayleigh scattering energy transfer).

**Table 7:** Different uses of CNMs in bioimaging applications.

Serial Number	Carbon Nanomaterial	Bioimaging Agent	Bioimaging Applications	Ref.
1	SWCNTs	Labelled recombinant thermo-stable *Luciola cruciata* luciferase (LcL).	Advanced powerful tool for in vivo imaging.	[195]
2	SWCNTs	SWCNT surfaces grafted with radical polymer produces brighter emission.	Bioimaging and biosensing in vivo in near-infrared region.	[196]
3	Carboxylated MWCNTs conjugated with polyelectrolytes (CPE)	MWNTs possess characteristic Raman vibration modes and CPE has optical properties; both provide fluorescence. Raman dual-imaging method.	Intracellular tracking and finding location of MWCNTs in in vitro and in vivo.	[197]
4	Carbon dots	Carbonization of sucrose with oil acid shows strong fluorescence and quantum yield.	Applicable in cell imaging.	[198]
5	Graphene oxide	GO nanosheets decorated with aptamer-labelled CdSe@ZnS QDs.	Potentially used in bio-imaging and cell-targeted drug delivery.	[199]
6	Fullerene	Fluorescent fullerene-coated mesoporous silica nanoparticles.	Fluorescent cell imaging and pH-sensitive drug release achieved.	[200]

TEG = Tetraethylene glycol.

**Table 8:** Different CNMs’ uses in vaccination.

Serial Number	Carbon Nanomaterial	Vaccinating Agent	Vaccine Applications	Ref.
1	SWCNTs	SWCNTs coupled with recombinant plasmid pcDNA-ORF149 (antigen).	Anti-KHV (Koi herpes virus) vaccine.	[214]
2	MWCNTs with OVA	MWCNTs (delivery system) with tumour-derived NY−ESO−1 (testis antigen).	Increased specific antibodies level in mouse model and delayed growth of tumor and prolonged survival.	[215]
3	Carboxylated MWNTs co-delivered with OVA, CpG and αCD40	OVA (antigen) and CpG and αCD40 (adjuvants).	Elevated T cell proliferation and IFN−γ secretion and enhanced antigen-specific CTL response reduce tumor growth and prolong survival.	[216]
4	Fullerene	Multihydroxylated fullerene as adjuvant and HCV recombinant proteins as antigens.	Induce humoral and cellular immune responses.	[217]
5	Carbon Dots	Fluorescent CDs as delivery system.	Provide access to trace antigen movement from the injected site to the lymph organs.	[218]
6	Graphene Oxide	Antigen-loaded alum-based adjuvant modifies GO nanosheets and induces humoral immune response the cellular immune response.	Powerful ability to raise cellular- and humoral-type immune response and improves cancer immunotherapy efficacy.	[219]

**Table 9:** Different CNMs used in PDT applications.

Serial Number	Carbon Nanomaterial	Photodynamic Therapy Agent	Applications	Ref.
1	SWCNTs	SWCNTs coated with Fe_3_O_4_ and CQDs conjugated to a DOX-loaded sgc8c aptamer act as both NIR ROS generators and drug loading carriers.	The multifunctional delivery platform should also carry chemotherapeutic agents for multifunctional imaged-guided PDT/PTT/ chemotherapy in cancer therapy.	[229]
2	MWCNTs	mTHPC (m-tetrahydroxyphenylchlorin) as photosensitizer.	Cancer treatment with combination of PDT and PTT.	[230]
3	SWCNHs	SWCNHs nanohybrid coated with TSCuPc and MPc, in which TSCuPc acts as PDT agent.	A 650 nm laser significantly increases the anticancer efficacy of combined noninvasive PDT.	[231]
4	Fullerene	DOX conjugated to C_60_ attached to a hydrophilic shell provides more stability and remote control through a laser (532 nm) for PDT.	Tumor targeted with “on-off” state for strengthening the treatment of cancer through combined therapeutic effects.	[232]
5	Nano-Graphene oxide (NGO)	NGO conjugated with ICG for PDT.	Enhanced antimicrobial and anti-biofilm activity against *E. faecalis*.	[233]
6	Carbon−silica nanocomposite (CSN)	CSN as PDT and as immunoadjuvant.	Harbors photothermal and photodynamic properties with potent antitumoral effects.	[234]

(MPc = metal phthalocyanines, TSCuPc = tetrasulfonic acid tetrasodium salt copper phthalocyanine, ICG = indocyanine green).

**Table 10:** Some foremost patents describe CNTs used in the delivery of therapeutics, imaging and in disease diagnosis.

Serial Number	Patent Number	Patent Description	Ref.
1	US20090062785	SWCNTs were attached to proteins (including, but not limited to, annexins) or peptides and formed protein-CNT complexes. Complexes bound to specifically to tumor cells rather than to healthy cells; the cells were used to diagnose and irradiate tumors at specific wavelengths. However, an immunostimulant was also administered to intensify the immune response of the patients against antigen released by tumor cells.	[235]
2	US20080227687	Proteins (annexins) were linked with SWCNTs to target cancerous cells, particularly tumor vasculature endothelial cells. To diagnose and destroy these tumors, a specific electromagnetic wavelength was employed.	[236]
3	US20100209479	MWCNTs were attached to chemotherapeutic agents, such as mitomycin C.	[237]
4	US20090136987	CNTs were loaded with contrasting agents and used as imaging agents for detection in a cell.	[238]
5	US20080193490	CNTs employed as drug delivery vehicles for cancer drugs. CNTs were encapsulated with therapeutic agents and surface modifications were performed with different functional groups.	[239]

## Data Availability

All datasets generated for this study are included in the article.
